# Reproductive Diseases in Farmed Rabbit Does

**DOI:** 10.3390/ani10101873

**Published:** 2020-10-14

**Authors:** Joan Maria Rosell, Luis Fernando de la Fuente, María Teresa Carbajo, Xosé María Fernández

**Affiliations:** 1 Cunivet Service, P.O. Box 518, 43080 Tarragona, Spain; 2 Departamento de Producción Animal, Facultad de Veterinaria, Avda. Profesor Pedro Cármenes s/n, Universidad de León, 24071 León, Spain; f.fuente@unileon.es; 3 Departamento de Medicina, Cirugía y Anatomía Veterinaria, Facultad de Veterinaria, Avda. Profesor Pedro Cármenes s/n, Universidad de León, 24071 León, Spain; mtcarr@unileon.es; 4 NANFOR, Aldea Loureiro, 40, 15980 Padrón (A Coruña), Spain; xm.fernandez@nutreco.com

**Keywords:** animal welfare, farmed rabbit, female infertility, reproductive disease

## Abstract

**Simple Summary:**

The domestic rabbit doe can have several reproductive diseases, including infertility. These were causes for consultations from rabbit producers. This led us to study the reproductive diseases of farmed does. To do this, we relied on visits to rabbitries, made since 1994. Low fertility, which in our case we considered less than 70% pregnancies (85.5% was the average observed on farms), plus an abortion rate equal to or greater than 2% of inseminated does, were the most frequent problems. Then, we scheduled work from 2014, through pregnancy checks with abdominal palpation. We recorded the results for each rebred lactating doe examined, along with data on her body and sanitary condition, and parity order. We found an effect of low body condition or diseases, such as mastitis, on infertility. Age also had an effect: first parity does were less fertile. Therefore, before servicing a doe, it is necessary to evaluate its condition, as well as various management practices (reproductive rhythm) or environmental factors (lighting).

**Abstract:**

In this study, we determined the occurrence of reproductive diseases in does on 1373 visited farms in Spain and Portugal, between 1994 and 2019. The retrospective information obtained was entered in a database classified as follows: apparent infertility (≤70% pregnancy rate), abortions (≥2% of serviced does), high fetal death risk at parturition (≥10%) or dystocia, amongst others. Infertility was the reason for 181 visits. The median of prevalence of apparent infertility in these cases was 35% (minimum to maximum: 25–90%) and the mean, 37.4%. We performed a prospective study to determine risk factors at the individual and farm level, with a second database corresponding to 2014–2019. We carried out pregnancy checks, assessed sanitary status and body condition, and recorded the age of 17,297 rebred lactating does on 142 farms. The median size of the farms was 800 does, and the examined cohorts, 350 does. Predisposing risk factors for infertility were observed: e.g., mastitis had an effect. During the 5-year study, we made a third database with the results from 190,508 does palpated by producers in a subset of 134 farms. In this case, the median of the prevalence of apparent infertility was 14.5% (minimum to maximum: 4.1–50%), which could be considered baseline occurrence when monitoring the theriogenology of rabbit doe farms. Reproductive rhythm was an enabling risk factor: does serviced ≤25 d postpartum were less fertile than at ≥32 d. We made a database with the body condition score (BCS) on a linear scale from 1 (emaciated) to 9 (obese). The pregnancy rate (PR) of underweight and borderline does (4/9) was 73.1%. The PR of overweight and borderline does (6/9) was 82.6% and those classified with a mean BCS (5/9): PR = 79.3%. We may infer that the optimum BCS for reproduction is 6/9, rather than 5/9. Some changes in female rabbit health and husbandry to improve reproductive performance and welfare are highlighted.

## 1. Introduction

The domesticated European rabbit belongs to a particularly fertile species (*Oryctolagus cuniculus*); rabbit does can combine lactation with a new pregnancy [1], although there are physiopathological limitations to this overlapping. Reproductive problems in farmed does might occur at any stage between service: mount or artificial insemination (AI) and weaning; nevertheless, the subject of this study is infertility. *Infertility is the state of a diminished capacity to conceive and bear offspring. In contrast to sterility, infertility is not an irreversible state* [2]. It therefore includes the male’s refusal to mount in farms using this method, non-pregnancies following service, embryo mortality, fetal resorption, or abortion; furthermore, it comprises premature birth (<30 d gestation), high fetal death risk at parturition, or weak non-viable kits. The International Rabbit Reproduction Group pointed out that for fertility, measuring aptitude for reproduction is usually defined by the kindling rate. Nevertheless, fertility can be estimated by the pregnancy rate [3]. In Spain, the pregnancy rate (pregnant females/palpated females) was 85.4% in 2018 and the kindling rate (parities/services) was 78.2% [4]. Therefore, the prevalence of real infertility on farms was 21.8%, whereas apparent infertility when pregnancy was diagnosed by abdominal palpation was 14.6%. Farmed does have to cope with different intrinsic production factors and the environment, which are generally favorable and occasionally harmful. Some risk factors predispose to apparent infertility in does, i.e., ≤70% pregnancies [5], such as diseases or body condition [6]. Environmental risk factors enable low fertility; for instance, lack of light [7], amongst others.

Reproduction is key to the wellbeing of does [8]. Obstetric processes, uterine disorders, or other causes of infertility, such as enteritis or heat stress, are more painful than infertility per se. Some female rabbit disorders affect the offspring, causing them to die before parturition, or only a small number of weak kits are born; this also has an effect on future post weaning [9]. Robustness is a core element in relation to health, diet, and reproductive efficiency in does [10]. Infertility is one of the main reasons for culling: the median monthly culling risk due to infertility was 0.9% [11]. Low fertility means that mount has to be repeated and housing for non-pregnant does is required; this results in 128% over-occupation of housing with nests [12]. On single batch farms, the daily cost of repetition at 11 d and parturitions every 42 days was 0.57 euros/day [13]. Controlling reproductive disorders on farms requires the work of skilled staff, for both manual tasks, e.g., pregnancy checks and quality data records, and decision-making [14]. Attending veterinarians assess reproductive diseases based on on-farm clinical features. They rarely perform slaughter checks because does are usually culled on the farm, unlike cows or sows, which are often taken to the abattoir [15].

Our aims were to (1) carry out a retrospective assessment of reproductive diseases in doe farms from 1994 to 2019 in Spain and Portugal, (2) describe apparent infertility and risk factors at the farm level by performing a prospective study from 2014 to 2019, and (3) estimate risk factors of infertility when the unit of analysis was the doe, in rebred lactating does, by carrying out pregnancy checks.

## 2. Materials and Methods

Our study period comprised 1st January 1994 to 31st December 2019. We obtained our information by carrying out 11,382 visits. They were as part of daily veterinary practice, routine health monitoring, and consulting activities on rabbit farms housing females in Spain and Portugal. Animal Care and Use Committee approval was not obtained for this study because data were obtained from rabbits raised under commercial conditions; farms fulfilled European, national, and regional recommendations and laws on animal welfare, food safety, and environmental protection.

### 2.1. Rabbit Farms, Doe Census, Breeds, and Lines

**Figure 1:**
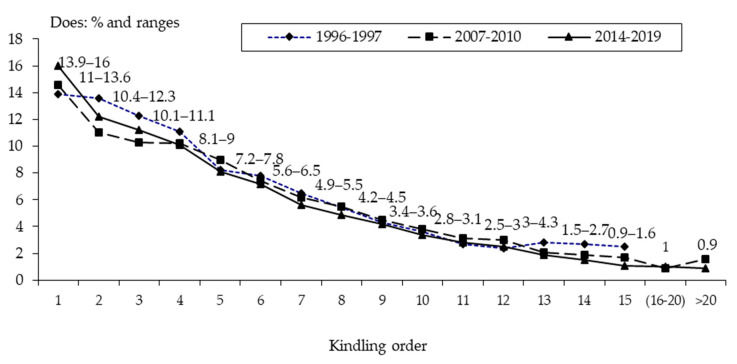
Descriptive age traits of 48,162 female rabbits, based on 10,422 examined does on 168 farms from 1996 to 1997 (…♦...). In addition, there were 20,985 does checked on 129 farms from 2007 to 2010 (…■…), and 16,755 does on 132 farms from 2014 to 2019 (…▲…). Data derived from Rosell and de la Fuente [20], Sánchez et al. [6], and the current study, respectively.

Data were gathered on 1373 visited rabbitries; 1283 in Spain and 90 in Portugal. Farms in Spain are inscribed in the official database *Registro General de Explotaciones Ganaderas* (REGA). According to the *Ministerio de Agricultura, Pesca y Alimentación* [16], the number of farms varied greatly throughout the 26-year study. In a preliminary paper, we provided a detailed explanation of the farms registered in the REGA database and those visited between 2001 and 2017 (Figure 1, in [17]), and those visited in Portugal. On each visit, we asked producers about their rabbit breeds and lines, doe inventories, i.e., females bred once or more, generally at ≥4.5 months old. In Spain and Portugal, the majority of food-producing rabbits belong to breeding companies, with the exception of New Zealand White rabbits (we examined does on two farms for laboratory and two for meat). In addition, we visited two doe farms with Rex rabbits, three doe farms for pets, not included in the current study, and also farms with non-selected “colored” breeds/lines [18].

### 2.2. On-Farm Management Procedures

We visited outdoor and indoor farms, with natural or mechanical ventilation; images may be seen on our website: https://www.cunivetservice.com/wp-content/uploads/2020/04/Poster.Types_.Rabbitries.Visited.1994.2019.23Apr2020_compressed.pdf. We recorded the type of service used on each farm and timing; rabbit producers use a fixed time service from ≤11 to 60 d after parturition. We also recorded the number of batches per farm, from 1 to 8; a batch is a group of rabbit does bred on the same day. A farm with one (single) batch, and AI on day 11 postpartum, had parities every 42 d, i.e., eight batches per year. The use of a single batch allows the “all in–all out” system to be used and housing to be cleaned and disinfected [19]. However, the median kindling-to-kindling interval per doe (KKD) was 51.4 d, i.e., 7.1 parities per doe per year [11]. On some farms, there was a single batch, on others ≥ 2 batches, or eight barns with a single batch per barn. For fixed AI timing, the KKD is a key indicator in farmed rabbit doe theriogenology.

### 2.3. Datasets and Cohorts

Information corresponding to the 26-year study period was grouped into four databases. In the first database, we included retrospective information on 292 visits (out of 11,382 visits in total) due to reproduction problems at the farm level (n = 176 out of 1373 farms). The second one included palpations on 142 farms between 28th November 2014 and 5th December 2019; this prospective study used cohort-level data and 481 cohorts. The cohort is a batch subgroup, i.e., a set of females served on the same day in a maternity barn or on a farm; in addition, does have another characteristic in common, e.g., physiological condition, or genetic line. Thus, a farm with does serviced 15 d before could have (a) first time AI-inseminated non-lactating does, (b) inseminated lactating does, (c) does that failed to conceive in the previous service, and d) non-rebred lactating does; in this example, there was a cohort at risk, i.e., rebred lactating does, which were our target. The relevance of the 2nd database is that it includes the results of palpations by producers in the cohorts that we sampled. Nevertheless, we only obtained the results of 427/481 cohorts with 190,508 females palpated by producers on 134 farms. The third database included individual assessments of 17,297 rebred lactating does, which we palpated on 142 farms, grouped into 481 cohorts. In this prospective study, we used female-level data. We included the age, body condition, and sanitary status of sampled gestating and empty does. The fourth database contained descriptive information on the sanitary status and body condition of 43,803 does and 270 farms, between 2006 and 2019. We used some of the data in a subset of 18,510 does in a previous paper [6]. Another subset of 17,297 does checked for pregnancy was used in this study. There were no repetitions of does between databases. Concerning does at risk, for instance, there could have been 1000 does on a farm, divided in two batches: one served 15 d previously (and then, able to be palpated) and another five days after parity; for this reason, we sorted sizes such as does per farm and does at risk (per cohort at risk).

### 2.4. Descriptive Traits of the Age of the Does

Figure 1 shows the descriptive distribution of ages based on 3 databases—1996–1997 [20], 2007–2010 [6], and 2014–2019.

The distribution of the 48,162 does was according to parturition number. Coutelet [21] states that the rate of first AI does was 14.1% per batch. According to our previous results, 70% of producers used the 42-day cycle; the interval between parturitions per doe was 51.4 d from the first service at 147 d, plus 31 d gestation. Thus, they were 6 months with one parturition, 8 months with two parturitions, 10 months with three, and so on [11]. Further comments will be made in Section 3.5.3.

### 2.5. Veterinary Visits to the Rabbit Farms

In this study, the clinical information on reproductive events was collected by Rosell in written records of observations during each on-farm visit. When classifying urgent visits, we only considered the main cause, e.g., myxomatosis or infertility, amongst others [5]. Farm managers were also asked about the service: if does were bred by AI or naturally mated, an AI center supplying semen, if any. In a previous study, AI was used on 85% (416/490 farms) of doe farms visited from 2006 to 2014; the semen came more commonly—87.5% (364/416 farms)—from AI centers [22]. We also asked the farm staff questions about procedures for preparing does for AI; for instance, the previous revision of sanitary and body condition (2 d before service or the same day), use of eCG, lighting schedules, or mother–litter separation, amongst other reproductive techniques [23].

### 2.6. Diagnostic Procedures Used

Pregnancy is diagnosed by abdominal palpation from 9 d following service, but mainly at 13–15 d to avoid errors. We palpated lactating does from 9 d after service. They were examined outside their individual housing. First, we observed the presence of clinical mastitis, rhinitis, ulcerative pododermatitis, and mange, following the protocol by Rosell [5]. All these criteria were classified as binary variables. We then assessed BCS, which enabled us to monitor energy balance. We used a linear scale from 1 (emaciated) to 9 (obese), 5 being the optimum [6]. Lastly, we carried out the pregnancy check; in addition, we measured the kappa index of concordance for diagnoses of pregnancy. We also assessed intrauterine abnormalities, e.g., clinical metritis, pyometra, or fetal mummification [24], and extrauterine abnormalities, e.g., ectopic pregnancy [25]. Following the criterion of Di Girolamo and Selleri [26], we included visits due to pregnancy toxemia in a high percentage of does; we excluded those due to mastitis, assessed in a previous study [17].

### 2.7. Statistical Analysis

Sample size (*n*) calculation was done with WinEpi software [27], using the following data: population at risk (*nl* lactating and rebred does), degree of expected confidence (95%), and expected prevalence (*p%*). When the examination was made, we calculated apparent prevalence of infertility with the population at risk, sample examined (*ne* does), infertile does found (*ni*), and degree of expected confidence (95%). We converted our anonymized raw data to Microsoft Excel 2007 (Microsoft Corp., Redmond, WA, USA). The analyzed outcome was apparent infertility. Independent variables were on-farm management and doe traits. Statistical analysis used in the dataset with 427 cohorts was by SAS [28], utilizing the General Linear Model(GLM) procedure. Statistical significance was indicated by a *p-*value < 0.05. Factors of variation with a possible effect on the dependent variable (fertility of the cohort) were estimated using the following model:

Y_ijklmn_ = μ + L_i_ + S_j_ + E_k_ + R_l_ + M_m_+ e_ijklmn_

where Y_ijklmn_ was the dependent variable, fertility on each visit-farm, μ was the mean, L_i_ was explained by the effect of the *i*th rabbit line (6 levels); S_j_, the effect of the *j*th type of service (2 levels: mount or AI); E_k_, the effect of the *k*th use of eCG, (2 levels: yes or no); R_l_, the effect of the *l*th AI timing (4 levels: 11, 18, 25, ≥32 d after kindling); M_m_, by the effect of the *m*th calendar month (12 levels); and e_ijklmn_ was the residual effect.

With regard to the dataset with 17,297 records included in 481 cohorts, we used the GENMOD procedure (SAS) to analyze individual fertility (+ vs. −), with logit link function, and the model:

Y_ijklm_ = μ + F_i_ + K_j_ + C_k_ + H_l_+ e_ijklm_

where Y_ijklm_ was the dependent variable, fertility on each checked female, μ was the mean, F_i_ was explained by the effect of *i*th farm (142); K_j_, by the effect of the *j*th number of kindling (9: 1 to 8, and ≥9); C_k_, the effect of *k*th body condition score (7: 2 to 8); H_l_, the effect of the *l*th sanitary status (7: healthy, rhinitis, mastitis, sore hocks, mange, uterine disorder, other); and e_ijklm_ the residual effect.

## 3. Results and Discussion

In this study, we assessed reproductive problems in farmed does. Some disorders are different to those affecting pet rabbits e.g., uterine adenocarcinoma [29], as farm does are younger [22]. We studied papers on rabbits reared for their meat, skin, wool, for laboratory purposes, or as pets, wild rabbits, and other animals. The rabbit is a so-called multi-purpose animal [30]; we agree with Bishop [31] in that this contributes to enhancing gaps in our knowledge. It means, in turn, that updated data may be clearly shown and farm staff are better trained and more motivated.

### 3.1. Descriptive Traits of the Farms Visited between 1994 and 2019

Between January 1994 and December 2019, we made 11,177 visits to 1283 farms in Spain, plus 205 visits to 90 farms in Portugal between 1999 and 2014. Some key traits of the 1373 farms visited are considered in Table 1.

These data correspond to the most specialized segment of farms with >200 does, according to the Spanish National Rabbit Breeding Survey [16]. During the 26-year study, the number of does per farm visited increased. In this study, we used the median for the description of the size of the farms. Thus, in 2000, the median was 580 does, although the average was 1000. The smallest farm visited in Spain (2000) had 75 does and the largest, 37,000 does; the difference in size is exceptional. On all of the farms visited during the 26-year period, does were kept in conventional individual housing, without elevated platforms. The change in management is evident; in Spain, AI was developed during 1981–1992 [32,33]. Mount was used in 1994 on 98% of farms visited, whereas AI increased by up to 90% from 2014 to 2019. After 1990, the reproduction rhythm evolved from intensive (<11 d postpartum) to semi-intensive (11 to 25 d) and extensive (≥32 d). In a preliminary study conducted in a group of 130 doe farms in Spain, the median kindling-to-kindling interval per doe was 51.4 d [11]. In France, it was 54.8 d with a parturition rate of 82.5% [21].

### 3.2. Reproductive Diseases in Does on Farms From 1994 to 2019

Reproductive diseases were underestimated due to the classification of urgent cases. For example, from 1994 to 2019, we made 111 visits to 50 farms with salmonellosis. There were often with high rates of abortions [34]; we classified these visits as salmonellosis.

#### 3.2.1. Description of Apparent Infertility on Farms from 1994 to 2019

Table 2 shows the retrospective results of apparent fertility or pregnancy rate and real fertility or kindling rate, from 1994 to 2018.

During the 26-year retrospective study, there was progress in genetics [35], as seen in the crossbreeding of rabbit lines selected for their reproductive or feeding efficiency [36]. In addition, the host’s resilience traits [10] have been developed accordingly. These factors consolidated the domestication of does, as a result of changes in reproduction (J.C. Domínguez Fdez.-Tejerina, personal communication).

Apparent infertility was 14.8% in 2018 and real infertility until parity was 21.6% (Table 2). These baselines may be included as doe welfare indicators [37] and in the theriogenology assessment of farms. Table 3 shows the classification of reproductive problems diagnosed on doe farms.

Each farm should have its own threshold. We assessed the results themselves or relatively; thus, ≤70% pregnancies are a problem, 88% in one batch, and 74% in the following one should also be analyzed. The median prevalence of apparent infertility in cases visited between 1994 and 2019 was 35% (minimum to maximum: 25–90%) and mean infertility 37.4%. Non-acceptance by the male in farms with mount could have been for pathological or physiological reasons: (1) rabbit females do not have a regular sexual cycle but estrus is more or less stable; this could explain refusal to mount per se or combined with age (worse in the first lactation) and low body condition score [39]. Refusal to mount could have been due to another reason, namely (2) pseudopregnancy. It is common in does that do not conceive after being served by sterile males, even mounted by other females, e.g., when housed together in groups [40]; the corpora lutea persist for between 6–7 and 15 d, and pseudopregnancy lasts 17 to 19 d [41]. On farms with ≥2 batches, does found non-pregnant with apparently good health and body condition were served in a batch 21 d later; the majority without treatment, although some producers used equine chorionic gonadotropin (eCG) again, and in other cases, prostaglandin PGF_2α_, 2 to 4 d prior to being rebred, respectively.

Apparent infertility is also due to embryo loss in the first 7 d post fertilization. Different risk factors participate in this stage, e.g., heat stress, the use of inappropriate antimicrobials in the first stage of pregnancy, age of the female, or genetic type [42]. We also diagnosed the following causes: (a) lack of water (e.g., 3 d after inseminating 1000 females: 50% positive). Secondly, (b) we found cases of inadequate lighting, e.g., as a result of a faulty clock. For this purpose, a minimum of 20 lux (lux meter) at eye level, 4 watts/m^2^ at a height of 2.25–2.50 m in daylight is necessary; when rearing is outdoors and the maternity room indoors, 40 lux and 8 watts are required (J-S. Ferré, personal communication). Producers use a lighting program of 16 h light–8 h dark, continuously or light flushing 7 days prior to AI [7]. We found infertility related with: (c) bad health due to non-specific causes (e.g., colibacillar enteritis-diarrhea) and specific causes, e.g., chlamydiosis. Other causes of infertility were: (d) misuse of pharmacological agents for reproduction control, e.g., 250 IU of eCG, 10 times the recommended dose (10% pregnancies were recorded). In another visit due to infertility, GnRH was not administered after insemination. We found adverse reactions to antimicrobials: e.g., florfenicol (subcutaneously, seen in 1995, and orally, in 2012). On 200 farms visited between 2012 and 2017, we observed that in 70% of cases, does were administered injectable antimicrobials at parturition [17]; when does survive dystocia, they should be treated because of the high risk of uterine infection [43]. We found: (e) toxicosis due to hypervitaminosis D and calcinosis; we visited 75 affected farms during 2010–2011 (diagnosed using Von Kossa staining by M. Domingo at the CReSA-IRTA, Barcelona). We found infertility and toxicosis as a result of feed contaminated by polyether ionophore antibiotics; the last visited case was in 2015—a 1000 doe farm which received feed with 11 ppm of monensin. We diagnosed infertility due to (f) underconsumption of feed (contamination with calcium carbonate). We observed infertility (g) due to the actions of inexperienced inseminators, an example of a bad human–animal relationship. We diagnosed (h) low fertility compatible with problems in the rearing period of future does: from weaning to 4.5 months old. This scope includes several features: (h.1) housing: individual, in groups, in separate rooms or with females. Secondly (h.2), feed is an important factor in adult females and young replacement does; young does can be feed with a low energy diet ad lib, or with a restricted female’s diet, and 4 d of flushing before AI, including feed [44], lighting [7], or both. Thirdly (h.3), during rearing, producers can apply several metaphylactic procedures and vaccination schedules. In addition (h.4), monitoring growth in young does should be assessed (e.g., >2.6 kg at 14 w). Fifth (h.5), we observed differences with age at first service: 4, 4.5, 5 1/4, or 6 months. Lastly (h.6), there were two options concerning management in the second AI: the does were served again at 11 d postpartum or at rest one batch, and so on. Information has been provided by different authors [45,46].

#### 3.2.2. Other Reproductive Diseases Diagnosed on Commercial Farms from 1994 to 2019

From 1994 until 2014, we necropsied 3666 does [11,22]. We examined 855 does/3666 (23.3%) with disorders of the reproductive system, including pregnancy toxemia and excluded mastitis [26]. This result is more similar to that obtained by Bertram et al. [47], with 236 cases of reproduction/854 does necropsied (27.6%), than Seidel [48], who found 1438 reproductive problems in 2100 necropsied does (68.1%). Images are available on the website link: https://www.cunivetservice.com/wp-content/uploads/2020/04/DisordersReproductionDoes.Images.23Apr2020_compressed.pdf.

##### Abortions

Abortions are a cause of infertility, besides early embryo death < 10 d after fertilization [49]; loss of conceptus during placentation—days 10–17 [50]; and late death at 17 to 24 d of pregnancy. Premature parturition occurs on days 28 and 29; the kits and others born weak on day 30 to sick or first kindling does are not viable on farms [51]. The abortion rate we use as a reference is 1% with regard to services; we consider ≥2% the interference level [5]. Of a total of 167 females that had aborted and were necropsied on 67 farms, 6.3% occurred between days 20 and 23 of pregnancy, 10% between days 24 and 26, 41.2% between days 27 and 29, and 42% between days 30 and 32, according to unpublished data from Rosell and de la Fuente [22]; this does not include visits to farms with a high rate of fetal death at parturition.

Throughout the 26-year study period, we diagnosed the following: infections, e.g., salmonellosis; on some farms there were >5% abortions. We found abortions due to intrauterine infections and fetal death on three visits due to chlamydiosis (confirmed in the laboratory). We also observed abortions and other serious disorders with listeriosis; outbreaks on several farms were compatible with feed distribution [52]. Listeriosis was controlled with subcutaneous injection of penicillin G benzathine, never orally administered, in agreement with Harknes et al. [53]. Infectious-specific myxomatosis was another cause of abortion [54]. We observed abortions due to non-specific and infectious causes; thus, 46 necropsied does/167 that had aborted also presented different degrees of pneumonia, 23/167 hemorrhagic septicemia, or 20/167 enteritis-diarrhea (plus 3/167 with mucoid enteropathy). The following agents were identified after microbiological analysis in the laboratory: *Escherichia coli*, *Pasteurella multocida*, and *Staphylococcus aureus* (opus cit.: [22]), also described by other authors [55].

Other causes of abortion on the farms we visited were (1) errors in management, e.g., does without water on day 23 of gestation—50.7% abortions; (2) toxicosis caused by polyether ionophores in feed. We observed reproductive problems, including abortion, due to mycotoxin poisoning, e.g., on a farm housing 350 does that had consumed feed containing 200 ppb zearalenone, twice the tolerated dose [56]. We found farms with high percentages of does with hypo- and hypervitaminosis A, and loss of conceptus. Vervueren [57] cites a case of abortion due to excess iodine, placental abruption occurring at 28 to 29 d. Finally, (3) abortion due to metabolic causes: there were cases of does having pregnancy toxemia/ketosis; we necropsied 96 does at 26 to 32 d gestation, seven of which had aborted. Seidel [48] analyzed 241 cases of abortion in a population of 2100 gestating does: 55% were caused by infection.

##### Kindling-Related Disorders of the Reproductive Tract

**Figure 2:**
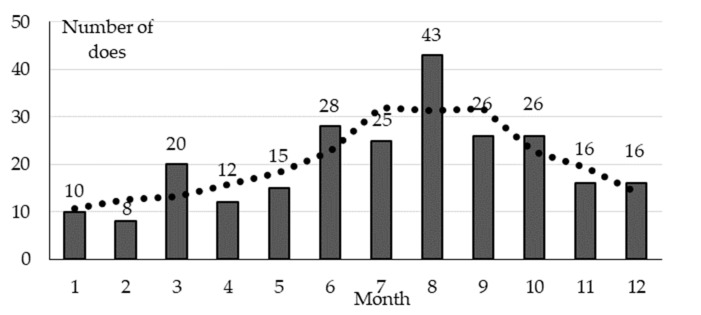
Monthly distribution of 245 necropsies with uterine torsion performed on 87 rabbitries from 1996 to 2014 and rolling average for three months (dotted line). Unpublished data derived from Rosell and de la Fuente [22].

Uterine torsion was the most frequent dystocia—67% of the 285 dystocias found between 2006 and 2014—and this was another reason for visiting a farm. Affected does die; only 6.1% of the necropsied does were euthanized. Our results showed that in 94% of cases, gestation was full term, with 6% at 28 to 30 d gestation, 64.5% at day 31, 21.7% day 32, and 7.8% day ≥ 33 d. We found cases of torsion of the vagina or uterine horn. The does were unable to deliver, although occasionally, torsion occurred after the doe had delivered some of her kits. The mean Monthly Mortality Risk (MMR%) and 95% binomial confidence interval (CI) in does were 0.20 (0.18–0.22%); 1.2 (1.01–1.39%) in the last week of pregnancy [22]. Frequency of torsion increased with age; of a total of 160 necropsies (at a known age) performed on 54 farms, from 1996 to 2019, 5 corresponded to 1st gestation, 8 to 2nd, 25 to 3rd, and 22 to 4th. It should be taken into account that in populations at risk, age decreased from first parturition onwards. Figure 2 includes the monthly distribution of all cases found on farms.

Season was an enabling risk factor of uterine torsion, with an increased incidence risk in summer (July to September). The monthly distribution of visits to farms was homogenous, as shown in a preliminary study on 13,467 visits made over a 30-year period (Table 2, in [54]). Lower frequency of movement in summer could increase this risk, as this occasionally occurs in bovines [58].

Other obstetric findings were vaginal, cervical, or uterine prolapse, with 0.21 MMR (0.19–0.25%). In 45 necropsies performed in 2006–2014, we found affected does on day 31 of gestation (82.2%), from day 26 to day 30 (11.1%), some (4.4%) the day after insemination, and 2.3% between 7 and 18 d after mount, according to unpublished data, derived from Rosell and de la Fuente [22]. We also diagnosed dystocia due to fetal macrosomia (1–2 oversized fetuses, e.g., in pregnancies with longer duration), or to a fetus obstructing the vaginal canal. Producers treat overdue does until day 32 with oxytocin SC, mean dose: 10 IU, i.e., 2 IU SC/kg BW. Proper treatment (uterine inertia vs. torsion) and dose are important as weights vary—mean BW was 4.72 ± 0.792 kg and median BW was 4.60 (minimum to maximum: 2.87–8.13 kg), as pointed out by de la Fuente and Rosell [18]. On visits to attend parturitions, we observed some live does with fetuses partially protruding from the vagina; we applied medical management with assisted vaginal delivery [59].

##### Uterine Conditions

Cases of metritis, pyometra and maceration, or fetal mummification were detected sporadically or within a wider range of infections: e.g., outbreaks of pneumonia caused by *P. multocida* or *S. aureus*, clinically diagnosed and identified in the laboratory (J.I. Badiola/CReSA-IRTA, personal communication). In EXOPOL (personal communication), analyses were carried out on 300 uterus, fetus, or both, samples from 101 farms between 2012 and 2019. *Staphylococcus aureus* predominated in 47 cases, *P. multocida* in 36, *Listeria monocytogenes* in 12, *E. coli* in 7, *Salmonella* spp. and other enterobacteria in 5, as well as mixed isolations of these agents or with *Streptococcus pyogenes*, *Pseudomonas aeruginosa*, and so on. Endometritis was not determined in our study; it causes subfertility [60] and is a common finding in pet does: 17.5% of uterine disorders [47]. In relation to disorders of the fallopian tubes, we diagnosed pyosalpinx [43] or persistent corpora lutea, on occasions related to uterine lesions. We found paraovarian cysts and, exceptionally, calcification during a serious outbreak of calcinosis (2010 to 2011). Lastly, from the on-farm biosecurity perspective, the spread of venereal (syphilis), specific (myxomatosis), or non-specific (pasteurellosis) diseases is fundamental [61] and should be emphasized when considering the advantages and drawbacks of AI.

##### Other Reproductive Disorders

Extrauterine-abdominal pregnancy was a secondary finding in necropsies performed on does in which the cause of death was pneumonia (3) or enteritis-diarrhea (2), or euthanized due to calcinosis (2). It was observed in culled does [25], as it is not usually fatal per se. Retained placenta occurred in exceptional cases (2). We did not carry out this diagnosis in vivo, as is the case in bovines [15]; however, Seidel [48] observed 188 does/2100. On some visits, we observed full-term aborted fetuses (>9 to 10%, Table 3), parturitions outside the nest, and the birth of weak kits. González-Redondo et al. [62] indicated parturitions outside the nest in primiparous does, or kits abandoned in the nest, coinciding with low temperatures. Heat stress (>30 °C) makes kindlings and kit survival difficult [8]. Lastly, we evaluated malformations on a farm during 2004 and 2005, where we checked 1356 parturitions and examined 12,654 live-born kits; the frequency of major external malformations was 1‰, less than the value of 6.6‰ in the study by Palmer [63].

### 3.3. Descriptive Traits of Farms Sampled between 2014 and 2019

Table 4 shows the traits of the 142 sampled farms from 2014 to 2019.

We made 432 visits to 142 farms where we palpated and examined 17,297 does, grouped in 481 cohorts. We also analyzed the results of palpations of all the does in each cohort, performed by the producers on 134 farms. The results of health assessment of the samples were normal. Mean prevalence of sore hocks was low: 6%, due to the fact that 87.7% of the examined does had footrests, slightly below the desired 100% [8].

### 3.4. Fertility and Variation Factors at Farm Level, on Farms Visited between 2014 and 2019

**Figure 3:**
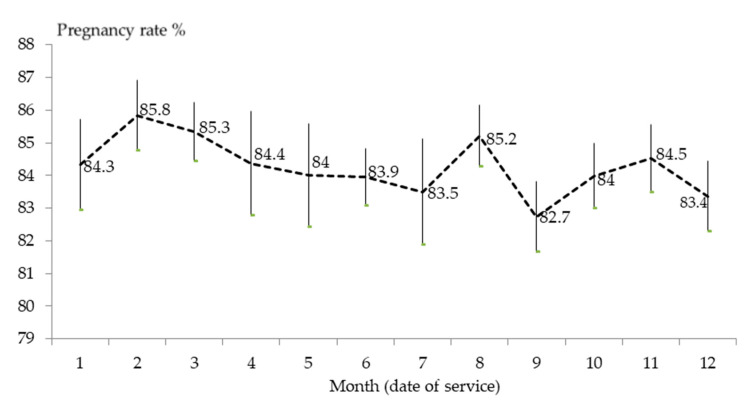
Monthly pregnancy rates and SEM, based on 427 cohorts with 190,508 does palpated by producers on 134 farms between 2014 and 2019.

Figure 3 shows the monthly distribution of fertility found by producers. The mean result was 84.32 ± 0.33% pregnancy rate and the median, 85.5% (minimum to maximum: 50–95.9%). Therefore, median prevalence of apparent infertility, determined by the pregnancy check (median: 15 d after AI), was 14.5% (minimum to maximum: 4.1–50%).

In this study, some variables had no effect on fertility. Firstly, there was no month or season effect. Only 5/142 farms did not have artificial lighting. On these five outdoor farms, we did observe seasonal infertility, with patterns of reduced acceptance and pregnancy, from October to early January. Additionally, some farms could have been affected by subfertility due to extreme temperatures, as well as relative humidity [21]. The breeding season is more favorable for does first serviced between December and March, than other times of the year [64]. Secondly, the eCG-based protocol, e.g., 25 IU administered at 48 h or 20 IU at 60 h prior to service, was not followed on 24 of the 134 farms (17.9%); 18 of these farms used AI ≥ 11 d, and 6, mount. According to our results, the use of eCG did not affect fertility either. This is interesting as far as rabbit welfare is concerned [37]. Thirdly, we did not observe any differences between the pregnancy rate in does serviced by mount (72 cohorts) and the 355 serviced by AI. In previous analyses [20], we found five percentage points in favor of mount.

Variables that did affect the pregnancy rate were, firstly, genetic type (*p* < 0.0001). We found significant differences between maternal lines, ranging from 83.38 ± 0.96% to 89.03 ± 1.28% pregnancies and females of heaviest lines, i.e., selected for growth, with 74.18 ± 2.33% pregnancies. In a previous study, we have shown a comparative insight into commercial lines and breeds, in a sample of 144,000 examined does [17]. The line effect [65] could be due to differences in feed efficiency and nutrient distribution [66], amongst other reasons. Secondly, AI timing also had an effect on fertility (*p* < 0.0001). The pregnancy rate in does serviced at 11 d was 82.61 ± 0.58%, 83.47 ± 1.06% at 18 d, 83.31 ± 0.94% at 25 d, and does serviced at ≥32 d were more fertile—87.47 ± 1.31% pregnant. This is an interesting result from the perspective of farm sustainability. García-García et al. [67] found no differences between 11 and 25 d. The reproductive rhythm is related to weaning age; they both interact on the reproductive outcome in does [68]. Nevertheless, kits born to the 16 doe farms serviced with AI ≥ 32d, remained with their mothers until they were sold, e.g., at 75 d (a farm with AI 60 d postpartum, 800 does in single batch).

### 3.5. Results Obtained from the Individual Examination of Does between 2014 and 2019

During the 5-year prospective study, we examined 17,297 does in a population of 202,209 females at risk, on 432 visits. There were 4289 empty does and 13,008 pregnant, examined in 481 samples (35 does per sample, not always randomly checked). However, the median of the pregnancy rate determined by producers was 85.6% (minimum to maximum: 50 to 95.9%) per visited cohort of does at risk, with a median size of 350 (Table 4). The mean pregnancy rate (84.3%) was similar to previous results in Spain [4]. In our study, infertility was influenced by several risk factors, as shown in Table 5.

The four analyzed factors were significant. The leading variable was farm, followed by the body condition score, and so on. The farm includes factors that influence fertility, e.g., the skill of the inseminators and the farm staff or environmental factors (temperature, relative humidity, and air speed), among others. These factors were not recorded but they had a high overall effect.

#### 3.5.1. Effect of Body Condition on Fertility

Body condition interacted with fertility, with a χ^2^ value of 429.89 (*p* < 0.0001). In 6308 does with a mean BCS (5/9), which in our opinion was optimum (we show images on our website: http://cunivetservice.com/docs/PosterVerona.June2008.pdf), the pregnancy rate was 79.3%. Pregnancies of 1810 does, which according to our evaluation, had a BCS of 6/9, i.e., overweight and borderline, increased to 82.9%. In 213 does with 7/9 (overweight, moderately fat), fertility remained at 82.6%, but in 38 obese does (8/9, plus one with BCS = 9/9), it decreased to 73.7%. In the 6955 does with a score of 4/9 (underweight and borderline), it decreased to 73.1%; in 1731 underweight and thin does (3/9), it reached 61.7%; and in 130 does with cachexia (2/9 plus six with BCS = 1/9), infertility was more prevalent—35.3% gestations. According to previous results [6], the BCS effect includes the feeding and sanitary status of does, besides BW [18], although the statistical models differentiate their effects. Some does had BCS = 9 at first and second parity. This depends on rearing and also unsuccessful mating in the first service; additionally, first gestation rabbit does are more susceptible to hepatic lipidosis, above all in second AI. Some attending veterinarians recommend culling does that are negative after first AI if the pregnancy rate in nulliparous does is > 90%. Based on these results, we should consider the possibility of 6/9 as the optimum BCS rather than 5/9, the theoretical mean, as pregnancy rates with a BCS of 6/9 and 7/9 were better.

#### 3.5.2. Effect of Sanitary Status on Fertility

Of the 17,297 examined does, 12,124 (70.1%) were apparently healthy. With regard to the health of the palpated does, the pregnancy rate of the 2670 with rhinitis did not vary in comparison with healthy does—77%. Pregnancy rate did decrease in the 393 does with mange (74%), 733 with sore hocks (72.2%), and above all, the 576 with clinical mastitis: 60%, that is, 22.1% fewer pregnant does than apparently healthy ones. We diagnosed 109 cases of metritis, pyometra, macerated, or mummified fetuses, even ectopic pregnancies. The incidence risk was 0.62% in the sampling of 17,297 does. On the 49 farms with apparent uterine or extrauterine abnormalities, the median incidence risk was 2.5 (minimum to maximum: 1.3–10%). Overall fertility in the 109 does was 43.1%. These disorders had different effects, causing sterility or subfertility, e.g., with fetuses in one uterine horn only, mummified fetuses, perhaps in the abdominal cavity, and fetuses in a new pregnancy. Four does/109 had had 19 to 21 parturitions and were almost three years old; prevalence of uterine adenocarcinoma until this age was <3% [47]. We did not diagnose any cases at necropsy, apart from one case of leiomyoma confirmed by N. Majó at the *Universitat Autónoma de Barcelona* [22].

#### 3.5.3. Age

On 90% of the farms we sampled, each doe had an individual record card. In this study, age had an effect on fertility, with a χ^2^ value of 68.14 (*p* < 0.0001). Apparent fertility in the 3225 1st parity and rebred does was 69.8%. The pregnancy rate in 2nd parity and 3rd AI does increased to 75.6%; in 3rd parity and 4th AI, 78%; 4th parity and 5th AI, 80%. From 6th parity and 7th AI on, fertility began to decrease, first to 77%, then in the 8th AI to 75%, in the 9th AI to 73%, and so on. Garreau et al. [69] observed a decrease in kindling rate, from 0.78 in the first AI to 0.69 in first parity and second AI and 0.73 in 3rd AI. With regard to age, in a more recent database of 43,803 does examined on 270 rabbitries between 2006 and 2019, the median was five parities (minimum to maximum: 1–39) and the mean, six (results not presented). Lenoir and Garreau [70] evaluated 20,000 does from 2005 to 2012, with a mean career of 5.7 AI; they used strict culling criteria, e.g., two negative AI. Farmed does do not have a long lifespan due to the removal risk, which includes 3% monthly mortality risk plus 7% culling risk, for different causes [11,22]. Age is a cause of culling; producers cull does, for example, when their record card is full, at between 11 and 14 services. However, this is not the case on all farms, as demonstrated by our true longevity record corresponding to a doe with 50 consecutive parturitions using AI at 11 d postpartum; on 8th July 2014, at over six years old, she delivered 11 live born kits. We agree with Savietto et al. [71], who recommend reducing the number of healthy does culled due to age, to increase maturity and achieve better sanitary stability on farms.

### 3.6. Limitations of the Study

The main sources of bias for the authors are related to the lack of diagnoses in necropsies, e.g., endometritis; in the case of producers, e.g., undetected abortions. There are also some record errors: e.g., pregnancy rates should be positive palpations out of the total number of does palpated, not services. We also found a number of misinterpretations: 75% of pregnancies is a bad result when mount is used; some producers underestimated the problem. Producers make few errors in palpations; however, there was a potential risk of bias due to differences in the stage of palpation. Misdiagnosis could have occurred in early stages (<11 d). In addition, there could have been cases of reabsorption before palpation at a late stage (>18 d). To assess errors due to early diagnosis, we palpated 177 does serviced 10 d before, on three farms in 2019. We checked them again 4 d later, with an error of 7.3%. In our opinion, this is a high rate in comparison with 3% [3]; false non-pregnancies are the problem.

We underestimated some results of the pregnancy check in rebred lactating does. We did not have information on the previous parturition, e.g., whether it was dystocic or whether the doe had aborted. Some producers will keep does that have aborted if their body condition is good. On the other hand, and in agreement with Fourichon et al. [72], we might have overestimated the results of the effect of some diseases on infertility, e.g., in that some risk factors were common to a particular disease and infertility. The sanitary status of a doe might change from the day of service, e.g., 11 d postpartum, to the pregnancy check, e.g., day 25 (11 + 14). Infertility can include the whole gestation period. A doe might conceive, maintain a pregnancy, and then, reabsorb the fetuses for different reasons; therefore, it is advisable to use real rather than apparent fertility. Our study does not include some aspects of all reproductive biotechnology protocols [73], or aspects of sanitary schedules (e.g., vaccination against myxomatosis). It would be interesting to focus more on primiparous does, separating them from older ones. Our pregnancy checks were carried out on lactating does; from the perspective of reproductive failure, non-lactating does would also be a target of interest.

## 4. Conclusions

This paper includes a retrospective study with the occurrence of reproductive diseases in farmed does between 1994 and 2019. Apparent infertility (≤70% pregnancy rate) was the most frequent reason for visits to farms. Therefore, we included a prospective study to determine risk factors involved in this disorder, at both the doe and farm level, from November 2014 to December 2019. The median of prevalence of apparent infertility was 14.5% in the 5-year study, which may be considered baseline. There were predisposing risk factors, e.g., diseases (mastitis) or the body condition score (target: 5 to 7/9), and enabling risk factors, e.g., the reproductive rhythm. Our study provides tools for assessing fertility in farm does and making further improvements and refinements. Reproductive problems occurred in primiparous does. Rearing of young does has been studied by several authors. Now, this scope deserves the approach and expertise of the stakeholders engaged in rabbit doe farming: producers, attending veterinarians, and technicians related with animal welfare, feed and farm equipment manufacturers, AI, and selection breeders’ centers.

## Figures and Tables

**Table 1:** Traits of doe farms visited in Spain and Portugal between 1994 and 2019.

Trait	Year							
	1994	1997	2000	2001	2003	2004	2006–2014 ^a^	2014–2019 ^b^
Number of visits	389	426	323	463	363	381	3278	438
Doe farms visited (n)	191	212	126	153	139	162	490	142
Farm size ^c^	350	450	580	600	600	604	769	800
Farms with AI ^d^ (%)	2	12	35	48	71	68	85	90
Days for service: <11, 11, >11 d (% farms)	NR	31,63,6	NR	17,71,12	10,65,25	4,79,16	0,75,25	0,70,30
One batch/farm (%)	NR	NR	NR	NR	30	30	46	48

^a^ From 1st January 2006 until 31st December 2014 [22]. ^b^ From 28th November 2014 until 5th December 2019 (present study, only in Spain). ^c^ Median number of does. ^d^ AI—artificial insemination. NR—not recorded.

**Table 2:** Description of pregnancy and kindling rates on farms in Spain from 1994 to 2018.

Trait	Year ^a^												
	1994	1996	1998	2000	2002	2004	2006	2008	2010	2012	2014	2016	2018
Number of farms	166	155	136	111	87	91	113	101	53	61	64	67	88
Farm size	391	473	617	661	826	832	921	914	697	684	653	648	657
Pregnancies % ^b^	82.1	82	81.4	82.3	81.1	81.9	81.7	84.3	83.2	85	84.5	84.9	85.2
Parities/services % ^c^	73.2	75	74.1	74.8	74.3	76.8	76.8	78.8	77	79.3	78.8	78.6	78.4

^a^ From 1994 to 2008 in Rosell and González [12]; from 2010 onwards, in Gullón et al. [4]. ^b^ Total pregnancies/palpations or apparent fertility. ^c^ Parities/services or real fertility.

**Table 3:** Classification of reproductive problems diagnosed as the main reason for 292 visits (out of 11,382 visits in total) to 176 farms (out of 1373 farms) in Spain and Portugal from 1994 to 2019.

Trait	No Visits	Target ^c^	Interference ^c^
Abortions ^a^	23	≤1%	≥2 %
Non-specific reproductive problem	29	NA	NA
Difference between % pregnancies and % parities	7	<7 points	≥10 points
Metritis, pyometra, fetal maceration	8	NA	NA
Others (dystocia, malformations)	6	NA	NA
Small litter size, weak kits	10	≥ 8.5	<7.5
Pregnancy toxemia/ketosis	3	NA	NA
Low male acceptance rate in farms with mount	10	>80%	<80%
Low pregnancy rate (infertility)	181	≥75%+	≤70%+
High fetal death risk at parturition ^b^	16	≤7%	>9 to 10%
Total	292	NA	NA

^a^ Percentage of abortions/services. ^b^ Coudert [38] suggested a target of 2% for parturitions with all fetuses stillborn. ^c^ Derived from Rosell [5]. NA—not applicable.

**Table 4:** Traits of the farms and cohorts sampled in Spain, between November 2014 and December 2019.

Trait	Mean	Minimum	Q2	Maximum
Number of rabbit does per visited farm	1033	145	800	6000
Number of does at risk examined per cohort (n = 481 cohorts) *	420	20	350	1750
Number of does examined per sample (n = 481 examined cohorts on 142 farms)	36.6	10	35	102
Days of abdominal palpation (producers)	14.3	11	15	27
% pregnancies (info. of 427 batches palpated by the producers on 134 farms)	84.3	50	85.6	95.9
Days of lactation of the examined cohort	31.4	18	30	74
Days of pregnancy check (veterinarians)	16.3	9	15	27
Rhinitis in the 481 examined cohorts %	16	0	12.5	86.7
Mastitis %	4.7	0	2.9	30
Ulcerative pododermatitis %	6.0	0	3.3	73.3
Manges %	3.0	0	0	94

* There were a total of 202,209 rebred lactating females on 142 doe farms, our target; 194,597 does were housed on 126 farms using AI.

**Table 5:** The GENMOD (logit link function) of the risk factors farm, number of kindling, body condition score, week of lactation, and sanitary status for the prevalence of apparent infertility in rabbit does. The study was based on physical examination of 17,297 does on 142 rabbit farms in Spain, between 2014 and 2019.

Factor of Variation	df	Chi-Square	*p* > Chi
Farm	141	1337.91	<0.0001
Number of kindling	9	68.14	<0.0001
Body condition score	7	429.89	<0.0001
Sanitary status	6	121.60	<0.0001
